# Hemoglobin During and Following a 4-Week Commercial Saturation Dive to 200 m

**DOI:** 10.3389/fphys.2019.01494

**Published:** 2019-12-06

**Authors:** Damian Łuczyński, Jacky Lautridou, Astrid Hjelde, Roxane Monnoyer, Ingrid Eftedal

**Affiliations:** ^1^Department of Circulation and Medical Imaging, Faculty of Medicine and Health Sciences, NTNU Norwegian University of Science and Technology, Trondheim, Norway; ^2^Faculty of Nursing and Health Sciences, Nord University, Bodø, Norway

**Keywords:** acclimatization, decompression, erythropoietin, erythropoiesis, heliox, mild anemia, hyperoxic saturation, relative hypoxia

## Abstract

Commercial saturation divers must acclimatize to hyperbaric hyperoxia in their work environment, and subsequently readjust to breathing normal air when their period in saturation is over. In this study, we measured hemoglobin (Hb) during and following 4 weeks of heliox saturation diving in order to monitor anemia development and the time for Hb to recover post-saturation. Male commercial saturation divers reported their capillary blood Hb daily, before, and during 28 days of heliox saturation to a working depth of circa 200 m (*n* = 11), and for 12 days at surface post-saturation (*n* = 9–7), using HemoCue 201+ Hb devices. Hb remained in normal range during the bottom phase, but fell during the decompression; reaching levels of mild anemia (≤13.6 g/dl) the day after the divers’ return to the surface. Hb was significantly lower than the pre-saturation baseline (14.7 ± 1.1 g/dl) on the fifth day post-saturation (12.8 ± 1.8 g/dl, *p* = 0.028), before reverting to normal after 6–7 days. At the end of the 12-day post-saturation period, Hb was not statistically different from the pre-saturation baseline. The observed Hb changes, although significant, were modest. While we cannot rule out effect of other factors, the presence of mild anemia may partially explain the transient fatigue that commercial saturation divers experience post-saturation.

## Introduction

Commercial saturation divers work in high-pressure environments, in which their bodies must acclimatize to a variety of physiological stress factors (Brubakk et al., 2014). While in hyperbaric saturation, the divers must adjust to breathing gases with an elevated partial pressure of oxygen (ppO_2_), with additional daily variations in ppO_2_ during work excursions in the bottom phase. When the period in saturation ends, they must readjust to breathing normobaric air. These transitions present substantial challenges to the oxygen homeostasis: the vital balance that sustains the body’s energy production while limiting oxygen toxicity (Semenza, 2010).

The body responds to changes in ppO_2_ by adjusting the activity of its oxygen-carrying blood cells: the erythrocytes. Physiological adjustment to hyperbaric hyperoxia in saturation diving is evident when the divers return to the surface. At that time, genes that control the production and activity of heme and hemoglobin (Hb) and the maturation of newly formed erythrocytes are widely downregulated (Kiboub et al., 2018b). The divers’ Hb protein levels are also low (Thorsen et al., 2001; Hofso et al., 2005; Deb et al., 2017; Kiboub et al., 2018a), causing oxygen transport capacity to be suboptimal for breathing normal air. During the first 24 h after decompression, a rise in erythropoietin (EPO) is an early indicator of readjustment to life at the surface (Hofso et al., 2005; Kiboub et al., 2018a). However, recovery takes time. In a recent study, commercial saturation divers reported high frequencies of headache and fatigue debuting shortly after decompression, with fatigues lasting 4.3 days on average (Imbert et al., 2018). This indicates that several days may be needed for oxygen transport capacity to recover, but no study to date has examined the time it takes for Hb levels to be restored after commercial saturation diving.

In this study, Hb was measured daily during a 4-week commercial saturation dive to circa 200 m of sea-water (msw) and for 12 days after, using a robust and reliable method. Based on prior reports of hematological responses to prolonged hyperbaric hyperoxia, we hypothesized that the divers’ Hb would decrease and aimed to monitor the potential development of anemia as well as the interval for Hb recovery after hyperbaric heliox saturation.

## Materials and Methods

### Ethics

Commercial saturation divers were studied during and after work assignments on the DSV Deep Arctic in the Mediterranean Ocean, August–December 2018. The study protocol was approved in advance by the Norwegian Regional Committee for Medical and Health Research Ethics (REK), approval number 2018/1184. All subjects were informed of the aim and scope and provided written consent before inclusion, and the experimental procedures were conducted according to the Declaration of Helsinki principles for ethical human experimentation.

### Subjects

The study subjects were experienced, male saturation divers; cleared for diving after a mandatory pre-dive medical examination and committed to saturation onboard the DSV Deep Arctic. Table 1 describes study subject characteristics. At the time of this study, the divers had already performed one or more saturations as part of the same diving campaign. Eleven divers participated in the study for the full duration of a 4-week saturation period; nine proceeded into a post-saturation follow-up. Due to limited supply of consumables, seven divers completed the 12th day of the follow-up.

**Table 1:** Study subject characteristics (*n* = 11).

	Age (years)	Saturation diving career (years)	VO_2_ max (L/min)*	Baseline Hb (g/dl)
Mean	42	13	50	14.7
(range)	(31–53)	(5–23)	(44–55)	(12.9–17.0)

* *VO_2_ max tests were done before the diving campaign began*.

### Hemoglobin Device Training and Hyperbaric Test

Prior to saturation, the divers prepared for the Hb protocol and performed pre-saturation Hb baseline measurements in the vessel hospital under researcher supervision, using an AA battery-operated HemoCue 201+ device with cuvettes and safety lancets (HemoCue AB, Angelholm, Sweden). To test its function in the hyperbaric heliox atmosphere, one HemoCue was compressed to 182 msw with the first dive team. There were no issues with Hb readings in saturation; a total of six Hb devices were used throughout the study without instrument failures or missed readings.

### Saturation Diving

After their pre-dive medical examination, the divers entered the pressure chambers and were compressed (blown down) over a period of about 6 h until they reached the storage depth of 182–200 msw. Saturation diving was conducted according to the contractor’s procedures; the 28-day saturation profile is illustrated in Figure 2A. During the bottom phase, the divers worked in teams of three on a fixed rotation; each shift lasted 12 h, with four overlapping shifts evenly distributed over the 24-h day. Each three-man team did one underwater work excursion (bell-run) per day to a depth of 192–210 m, 7 days per week. A bell-run lasted 8 h maximum, during which two divers spent a maximum of 6 h in the water for work interrupted in the middle by a 30-min hydration break inside the bell. The third diver (bellman) remained in the bell for support. The bellman positon rotated, with each diver taking his turn as bellman every third day. At the end of the bottom phase, the divers were decompressed back to atmospheric pressure over a period of 8 days following the contractor’s protocol. In the bottom phase, the ppO_2_ was kept at 40 kPa in the pressure chamber and increased to 60–80 kPa during bell-runs. During decompression, the ppO_2_ was increased to 50 kPa until reaching 13 msw; from there up until surface the ppO_2_ was gradually reduced from 23 to 21%. After surfacing the divers remained on a 24-h watch for signs and symptoms of decompression sickness (bendwatch) before they left the vessel.

### Hemoglobin Measurements

The divers measured their Hb daily at the start of each work shift, after hydration but before breakfast. Sampling of capillary blood from the third or fourth fingertip was done according to the HemoCue201+ manual, using the third drop of blood from the punctuation site to fill the cuvette. The procedure was repeated in cases of visual bubbles or if the cuvette was incompletely filled. Hb readings were done within 10 min of blood sampling, and each diver used one Hb device and cuvette batch throughout the study. Readings done within the pressure chambers or on land after saturation were reported in writing to saturation control or directly to one of the researchers.

### Hemoglobin Analysis

Statistical analysis of the Hb data was done in IBM SPSS Statistics software Version 25.0. (SPSS, RRID:SCR_002865). Hb values deviating >1.5× from the lower or upper quartile were categorized as outliers, leading to exclusion of 5 of 404 data points. For the 399 data points included, normal distribution was confirmed by boxplot and Kolmogorov-Smirnov test. Homogeneity of variances was assessed by Levene’s test, which assumes homogeneity for *p* > 0.05. For this dataset *p* = 0.318. One-way repeated measures ANOVA with Bonferroni *post hoc* adjustment for multiple comparisons was used to assess differences in Hb concentration within the different phases separately: bottom phase, decompression and post-saturation. A linear mixed model with Bonferroni adjustment was used to assess the difference between Hb at pre-saturation baseline, the mean lowest value post-saturation (day 33) and the last day of follow-up (day 40). If Mauchlyʼs test showed that the assumption of sphericity was violated, a Greenhouse-Geisser correction was applied. Differences were considered significant at *p* < 0.05. Hb levels were graded according to WHO’s classification for adult males (Beutler and Waalen, 2006; WHO, 2011); 13.7–17.5 g/dl were considered normal, and 11.0–13.6 g/dl were considered mildly anemic.

## Results

**Figure 1:**
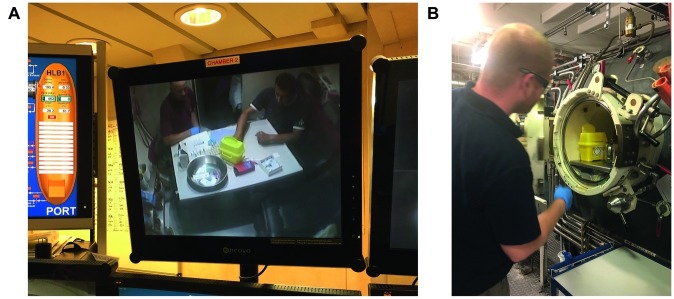
**(Panel A)** Divers in saturation onboard the DSV Deep Arctic measuring Hb on a battery-operated HemoCue 201+ device (red box on the table in Chamber 2). Whereas the Hb device and unused consumables remained in the hyperbaric chamber for the duration of the saturation, waste from the procedure was transported out *via* a decompression lock **(Panel B)**.

**Figure 2:**
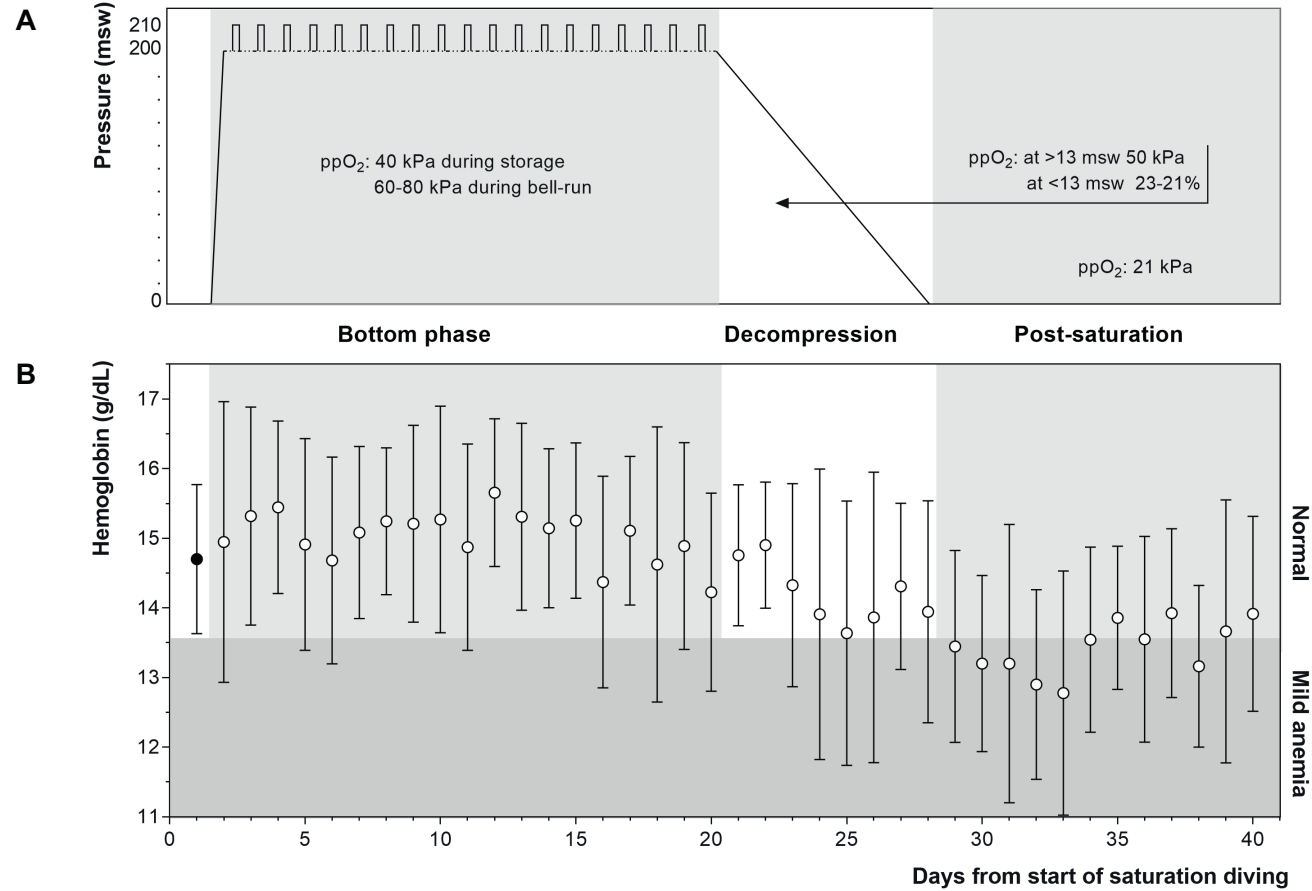
**(Panel A)** Heliox saturation profile. Daily bell-runs for work excursions are indicated by vertical bars on the bottom phase profile. During the bottom phase, ppO_2_ was kept at 40 kPa in the pressure chamber and increased to 60–80 kPa during bell-runs. In the decompression phase, ppO_2_ was initially raised to facilitate the elimination of helium, and gradually lowered during the final 13 m toward the surface to avoid explosion hazards. **(Panel B)** Daily Hb levels (mean ± SD) at baseline (filled circle), and during (*n* = 11) and following (*n* = 9–7) 4 weeks of commercial saturation diving (open circles). Hb ≤ 13.6 g/dl was graded as mild anemia.

**Figure 3:**
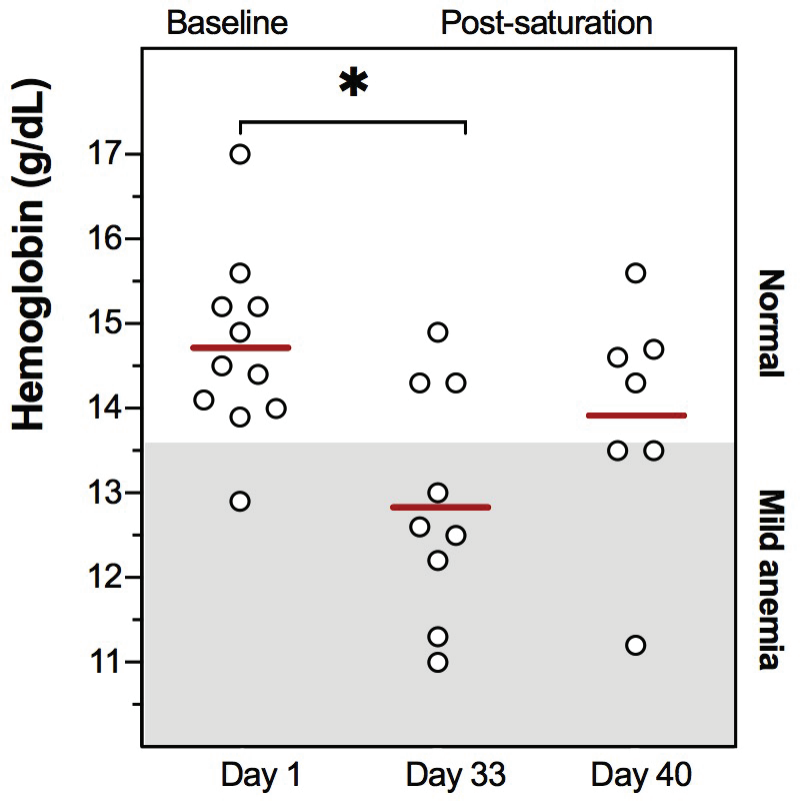
Individual Hb values at pre-dive baseline (Day 1), 5 days post-saturation (Day 33), and 12 days post-saturation (Day 40). Means are shown as red lines. The range for mild anemia (Hb ≤ 13.6 g/dl) is indicated in gray. ^*^p = 0.028.

Commercial saturation divers reported their Hb daily throughout and following a 4-week work period in heliox saturation. The practical set-up for Hb measurements inside a hyperbaric living chamber is shown in Figure 1. The dive profile is illustrated in Figure 2A, with bell-runs and ppO_2_ during and after saturation. The daily progression of Hb is presented in Figure 2B. The divers started their saturation with a normal Hb of 14.7 ± 1.1 g/dl (mean ± SD), and there was no significant change in Hb within any phases of the study. Hb remained in normal range through the bottom phase. There was a drop during the decompression phase, with Hb reaching levels of mild anemia (≤13.6 g/dl) the day after the divers’ return to surface. Five days post-saturation, Hb was significantly lower than the pre-saturation baseline (Figure 3, 12.8 ± 1.8 g/dl, *p* = 0.028), before gradually reverting to normal after 6–7 days. After 12 days of post-saturation follow up, Hb was still below the baseline but the difference was not statistically significant.

## Discussion

In this study, Hb was measured daily through 4 weeks of commercial heliox saturation diving to approximately 200 msw and a follow-up period after, using portable Hb devices operated by the study subjects themselves. While Hb remained normal during the bottom phase of the dive, a drop during the decompression phase progressed into mild anemia in the initial days after saturation. Return to normal Hb occurred within about 1 week. However, after 12 days of post-saturation follow-up, Hb was still slighty lower than pre-saturation.

Several studies have reported altered hematological status and Hb reduction after saturation diving (Thorsen et al., 2001; Smith et al., 2004; Hofso et al., 2005; Deb et al., 2017; Kiboub et al., 2018a). In our study, Hb remained unaltered through the bottom phase. In a prior report of experimental saturation to 240 msw, EPO was decreased 2 days into the dive (Hofso et al., 2005). This might be expected to cause Hb to fall, but we found no such fall. However, the divers in both studies experienced daily ppO_2_ variation during bell-runs. It is conceivable that these ppO_2_ changes are perceived by the body as bouts of relative hyperoxia/hypoxia, triggering EPO production after each bell-run and promoting erythrocyte production (erythropoiesis), equivalent to the theory of the normobaric oxygen paradox (Balestra et al., 2006).

In our study, Hb fell during the decompression phase. During this phase, living chamber ppO_2_ was increased from 40 to 50 kPa in order to limit the risk of decompression sickness during helium wash-out. In the last 13 m, ppO_2_ was gradually reduced to 21 kPa. A constant, high ppO_2_ would be expected to downregulate EPO and thus lower erythropoiesis and Hb. This is consistent with our observation. The drop in Hb continued over the first 5 days after the decompression, before it gradually returned to normal. Breathing air at the surface after saturation causes EPO to rise (Hofso et al., 2005; Kiboub et al., 2018a), stimulating erythropoiesis. However, erythropoiesis is a complex process; the maturation of new erythrocytes has been shown experimentally to take upwards of 1 week (Ni et al., 1999). In our study, the divers’ Hb levels were restored to normality 6 to 7 days after resurfacing. At that time, their Hb levels were still rising.

The detection method affects the outcome of Hb measurements. In this study we used battery-operated HemoCue201+ devices. Venous blood analyzed on an automated analyzer or cell counter is established best practice, and capillary blood has been shown to give higher Hb readings (Bahadur et al., 2010). However, the HemoCue is considered to provide acceptable data for the exclusion of anemia in blood donors, and for survey of anemia prevalence in a population (Patel et al., 2013). All in all, measuring Hb in capillary blood on a HemoCue is safe and reliable (Daves et al., 2016), and the device is robust and easy to operate. For this study, it should be noted that the HemoCue is calibrated for use in normobaric air, and there was no prior experience of its use in a hyperbaric heliox atmosphere. We noticed that the readings after blow-down appeared higher than those done before the divers entered the hyperbaric chambers, but the difference was within the range expected for this Hb device (Rappaport et al., 2017; Castren et al., 2018).

There are several limitations to the interpretation of the results of this study. First, we made no direct assessment of the divers’ hydration status. Dehydration is a known issue in saturation diving, where diuresis through compression and at high ambient pressure (Goldinger et al., 1992) and during water immersion (Epstein, 1976), and sweating in hot-water suits (Hope et al., 2005) contribute to fluid loss and consequently to reduced plasma volume (Jimenez et al., 1999). To counteract dehydration the divers were instructed to drink before blood sampling. They performed physically demanding work throughout the bottom phase, and significant fluid loss would be expected to weaken the divers physical work capacity (Saltin, 1964; Craig and Cummings, 1966); therefore, dehydration was unlikely to be a major issue. But it should be noted that reduction in plasma volume after saturation diving has been reported (Deb et al., 2017), indicating that we cannot rule out dehydration that might skew the Hb towards higher values (Carraro et al., 2015).

As we did not assess the divers’ full hematological status we cannot conclude whether the changes in Hb were caused by a lower blood count, a drop in Hb within the erythrocytes, or reduced erythrocyte size.

The diving was active around the clock, with a new dive team starting their shift every 6 h. It was impossible to control for effects of time of day with the number of subjects enrolled. However, as mature erythrocytes contain no nuclear DNA, they have no “clock genes” to induce circadian variation in their activity. Although alternative pathways for rhythmic control exist (Henslee et al., 2017), we have no reason to believe that the Hb measurements were affected by time of day.

Blood sampling in the hyperbaric chambers was challenging at times. Blood vessels may contract during simulated air dives (Polkinghorne et al., 1989) and HBO treatment (Vucetic et al., 2004), and on some instances the sampling under pressure had to be repeated several times due to difficulties accessing a capillary to collect blood. No readings were missed, and even though we cannot rule out mechanical hemolysis during blood collection (McCaughey et al., 2017), this has been reported to not interfere with capillary Hb measurements (Mokhtariye et al., 2019).

Finally, the number of divers participating in the post-saturation follow-up was limited by the number of Hb devices available to send home with them, and the time for follow-up was limited by the consumables they received. We ended the Hb analysis 12 days post-saturation with data from seven divers, but some divers continued to report Hb up to 18 days post-saturation. The data from these last days showed Hb to rise to pre-saturation levels, but the data were omitted from analysis due to insufficient power.

The results of our study raise questions of how to best protect the divers’ hematological status and Hb recovery after saturation. Dietary supplements are commonly used to improve Hb levels. Nutritional recommendations in saturation diving have been extensively reviewed by Deb et al. (2016), and although their review focuses on nutrition during saturation the same recommendations may be applied to the period after saturation. They recommend supplementation with vitamins B9 (folate) and B12 (cobalamin), which are vital for erythropoiesis and used in prevention and treatment of physiological anemia (Koury and Ponka, 2004; Koury, 2016). Erythropoiesis also requires bioavailable (ferrous) iron, but since iron is reported to accumulate in saturation divers’ bodies (Thorsen et al., 2001; Hofso et al., 2005; Zwart et al., 2012) additional supplements have not been recommended (Deb et al., 2016). Possible amendments for the inactivity in the decompression phase are also worth considering. After the workload the divers experience during bell-runs, the lack of physical activity during decompression may cause a decline of erythrocyte activity. This might be counteracted by effects of exercise training (Mairbäurl, 2013). A set of light exercise activities suitable for the confined space in the pressure chambers, possibly coupled with means to encourage activities using e.g. virtual reality tools, might alleviate the return to the surface while promoting erythropoiesis.

## Conclusion

After a four-week saturation dive to ca. 200 m depth, the divers developed mild anemia within the first 24 h of surfacing. Hb reverted to normal within circa 1 week. The observed Hb changes, although significant, were modest. While we cannot rule out effects of other factors, the presence of mild anemia may partially explain the transient fatigue that commercial saturation divers experience post-saturation.

## Data Availability Statement

The datasets generated for this study are available on request to the corresponding author.

## Ethics Statement

The studies involving human participants were reviewed and approved by Norwegian Regional Committee for Medical and Health Research Ethics (REK). The patients/participants provided their written informed consent to participate in this study.

## Author Contributions

DŁ and IE designed the study and managed data collection. DŁ and AH conducted the statistical analysis. JL, AH, and IE wrote the manuscript. RM reviewed the literature. All authors reviewed and approved the final version.

### Conflict of Interest

TechnipFMC sponsored helicopter transfers and boarding on the DSV Deep Arctic for DŁ and IE.

The remaining authors declare that the research was conducted in the absence of any commercial or financial relationships that could be construed as a potential conflict of interest.
